# Understanding personalized dynamics to inform precision medicine: a dynamic time warp analysis of 255 depressed inpatients

**DOI:** 10.1186/s12916-020-01867-5

**Published:** 2020-12-23

**Authors:** K. Hebbrecht, M. Stuivenga, T. Birkenhäger, M. Morrens, E. I. Fried, B. Sabbe, E. J. Giltay

**Affiliations:** 1 Collaborative Antwerp Psychiatric Research Institute (CAPRI), Department of Biomedical Sciences, University of Antwerp, Stationsstraat 22c, 2570 Duffel, Belgium; 2 University Psychiatric Hospital Duffel, VZW Emmaüs, Duffel, Belgium; 3 Department of Psychiatry, Erasmus Medical Center, Rotterdam, The Netherlands; 4 Department of Psychiatry, Leiden University Medical Center, Leiden, The Netherlands; 5 Department of Clinical Psychology, Leiden University, 2300 RA Leiden, The Netherlands

**Keywords:** Major depressive disorder, Routine outcome monitoring, Cluster analysis, Symptom dynamics, Inter-individual variation, Intra-individual variation, Symptom trajectories

## Abstract

**Background:**

Major depressive disorder (MDD) shows large heterogeneity of symptoms between patients, but within patients, particular symptom clusters may show similar trajectories. While symptom clusters and networks have mostly been studied using cross-sectional designs, temporal dynamics of symptoms within patients may yield information that facilitates personalized medicine. Here, we aim to cluster depressive symptom dynamics through dynamic time warping (DTW) analysis.

**Methods:**

The 17-item Hamilton Rating Scale for Depression (HRSD-17) was administered every 2 weeks for a median of 11 weeks in 255 depressed inpatients. The DTW analysis modeled the temporal dynamics of each pair of individual HRSD-17 items within each patient (i.e., 69,360 calculated “DTW distances”). Subsequently, hierarchical clustering and network models were estimated based on similarities in symptom dynamics both within each patient and at the group level.

**Results:**

The sample had a mean age of 51 (SD 15.4), and 64.7% were female. Clusters and networks based on symptom dynamics markedly differed across patients. At the group level, five dynamic symptom clusters emerged, which differed from a previously published cross-sectional network. Patients who showed treatment response or remission had the shortest average DTW distance, indicating denser networks with more synchronous symptom trajectories.

**Conclusions:**

Symptom dynamics over time can be clustered and visualized using DTW. DTW represents a promising new approach for studying symptom dynamics with the potential to facilitate personalized psychiatric care.

**Supplementary Information:**

The online version contains supplementary material available at 10.1186/s12916-020-01867-5.

## Background

Depression is defined by its symptoms (such as a sad mood and insomnia) that are correlated with each other. The dominant explanation in the field has been that these relations stem from a shared causal origin, a perspective termed the *common cause framework* [1, 2]. The contemporary conceptualization for major depressive disorder (MDD) is similar to that of other medical conditions in that it assumes all observable depressive symptoms are caused by an underlying disease construct [1, 3]. In research, symptoms are usually added up to sum scores, and thresholds are used to indicate case status. This approach assumes that symptoms are equivalent, causally independent, and roughly interchangeable indicators of the underlying disease construct [4]. This conceptual framework has dominated depression research over the past decades: the inclusion criteria in research studies were based on the syndromal DSM diagnoses of MDD, and the unweighted sum scores of depression rating scales were used as a measure for severity and treatment response [5] (e.g., Hamilton Rating Scale for Depression [HRSD] [6] and Montgomery-Åsberg Depression Rating Scale [MADRS]) [7]. However, years of research have shown slow progress in the search for the underlying risk factors and biomarkers of the unitary construct MDD [4]. The need for a new, scientifically sound approach for conceptualizing depression is warranted.

Increasing evidence points towards the multidimensional character of MDD with a high degree of symptomatic variability between and within patients [4, 8]. Individual symptoms are mutually interacting and causing each other [9], and they have different risk factors [10, 11], underlying biology [11–13], psychosocial impact [14], and course trajectories [5]*.* Recent years have therefore seen a shift in the conceptualization of depression towards a *network perspective* where the depressive syndrome is hypothesized to stem from mutual causal relations among components of the system, such as depression symptoms [9, 15]. Furthermore, patients manifest specific depression symptom profiles with preferential responses to different treatments*.* Consequently, there is increasing recognition of the importance of investigating individual symptoms and their timely evolution, both within individual patients and in groups of patients [16, 17]. This is also in line with the aims of the Research Domain Criteria (RDoC) project to deconstruct psychiatric disorders by analyzing the dynamics (e.g., symptom trajectories over time) that lie at their basis [18, 19].

Several factor analytic studies of the HRSD-17 have tried to tackle the symptomatic diversity of MDD by means of identifying homogeneous symptom groups within MDD. Although there was evidence for a “general depression” and “insomnia” factor, the overall results were inconsistent, with reported factors ranging from two to eight [20–23]. Furthermore, factors seemed to change over time [24] and were poorly generalizable to other populations. Hierarchical cluster analysis is another statistical method to decompose MDD into homogeneous symptom groups, and comparable results (“general depression,” “insomnia”) have been found with this approach [25, 26]. Network analysis is a more recent approach that expands further on studying the symptom correlations by investigating the influence of symptoms on each other [9]. Both factor and network analyses were mostly conducted on cross-sectional data, and consequently, they did not take the temporal dynamics of symptoms into account. Furthermore, both techniques almost exclusively studied aggregated patient data without studying the intra-individual symptom heterogeneity.

Routine outcome monitoring (ROM) entails the collection of clinical data at baseline and at regular time intervals thereafter in order to monitor disease severity as well as the clinical course during treatment. ROM may provide feedback to both the clinician and the patient and enable “patient-centered research” [27, 28]. Time-series ROM data enable the capturing of dynamics of symptoms over time using dynamic time warping (DTW). DTW is a widely used statistical algorithm [29, 30], though not yet in psychological and psychiatric research. It is an effective clustering strategy for time-series data across a broad range of application domains [31]. Examples of biomedical applications are speech recognition [16], gait pathology [32–34], and electro-cardiogram analysis [35]. The DTW approach could be well-suited to cluster individual symptoms based on the temporal features that they share, using ROM or ecological momentary assessment EMA [36] time-series data.

In this study, we utilize depression symptom data from a clinical ROM regime, every 2 weeks, of 255 depressed inpatients and present the first implementation of DTW time-series analysis on depression symptom trajectories. This paper is built upon a dual structure in which the DTW analysis is introduced both for intra-individual (i.e., idiographic) and inter-individual (i.e., nomothetic) analysis [37]. In the idiographic analysis, we aim to assess the dynamics and covariation of changes in symptoms over time within each individual patient with two or more assessments and to estimate the symptom clusters and networks within each patient. In the nomothetic analysis, we aim to study the aggregated dynamics of individual symptoms to yield systematic patterns across patients.

## Methods

### Sample and setting

From the original study sample of 276 consecutive patients (i.e., included in the cohort study in the order that they were admitted), 21 patients had only one HRSD-17 measurement due to a short hospitalization period or refusal to participate, yielding 255 (91.6%) patients included in the current analysis. Thus, we included 255 adult patients consecutively admitted to a tertiary psychiatric hospital in Duffel, Belgium, and fulfilling the MINI-Plus diagnosis, based on the DSM-IV criteria, of a depressive episode as part of an MDD or bipolar disorder (BD). In order to obtain a representative sample of depressed participants, exclusion criteria were minimal. We did not include patients with (comorbid MINI-Plus) psychotic disorders (including schizoaffective disorder) or with a dependency on alcohol or drugs within 12 months prior to hospitalization. Moreover, patients with insufficient mastery of the Dutch language were not included.

### Treatment

Inpatients received treatment as usual which was based on evidence-based guidelines and consisted of pharmacotherapy, (group) psychotherapy, or a combination of both. These guidelines for diagnosis and treatment were formulated by the Dutch Association of Psychiatry, often in association with the associations of psychology and general practitioners (www.trimbos.nl, www.nvvp.net). Psychotropic medication at baseline was coded into five dichotomous variables: antidepressants, mood stabilizers, antipsychotics, benzodiazepines, and stimulants. Response was defined as a ≥ 50% reduction of the HRSD-17 compared to the baseline assessment. Remission was defined as scoring ≤ 7 on the HRSD-17.

### Measurements

The present study was part of a larger follow-up study investigating the feasibility of ROM in the University Psychiatric Centre in Duffel [38]. ROM were done at baseline and every 2 weeks thereafter during the clinical admission which lasted from 2 weeks to 16 months. ROM consisted of a test battery assessing overall mental well-being, quality of life, and mood (including the Hamilton Depression Rating Scale-17). The data presented in this article represent the collected data from the period April 2015 through February 2018.

The HRSD-17 consists of 17 items on a Likert scale, ranging from either 0 to 4 (for 9 items) or 0 to 2 (for 8 items). The internal reliability of the HRSD-17 is adequate with most studies reporting a Cronbach’s alpha of ≥ 0.70. It has a good retest and interrater reliability (above 0.80) when assessed over an interval ranging from 1 to 30 days [21]*.* The Omega and Cronbach’s alpha in our sample of 255 patients at baseline were only 0.49 and 0.52, respectively*.* The Cronbach’s alpha improved over time with a score of 0.74 after 2, 0.77 after 4, and 0.79 after 6 weeks. The total score ranges from 0 to 52, and higher scores indicate greater severity, but in the present study, we focus on the trajectories of the 17 individual items only. In order to improve interrater reliability, Hamilton Depression Rating Scale training sessions were organized every 3 months among the in total 6 assessors, during which video-recorded interviews with patients were rated and discussed to reach consensus. In total, they conducted 1480 HRSD-17 assessments in 255 patients, with an average of 5.8 assessments per patient.

### Statistical analysis

DTW is an approximate pattern detection algorithm that can measure the similarity between two time-series. It uses a dynamic (i.e., stretching and compressing) programming approach to minimize a predefined distance measure (e.g., Euclidean distance), in order for the two time-series to become optimally aligned through a warping path. The “optimal” alignment minimizes the sum of distances between the aligned elements. The “dtw” (version 1.20.1), “pheatmap” (version 1.0.12), “parallelDist” (version 0.2.4), and “qgraph” (version 1.6.2) packages for the R statistical software were used (R version 3.6.0; R Foundation for Statistical Computing, Vienna, Austria, 2016. URL: https://www.R-project.org/).

The idiographic approach per patient was followed by a nomothetic approach to study the depression symptom patterns both within individual patients and in the whole sample of 255 patients. The subsequent methodological steps and statistical methods are described below.

### Intra-individual approach

**Fig. 1:**
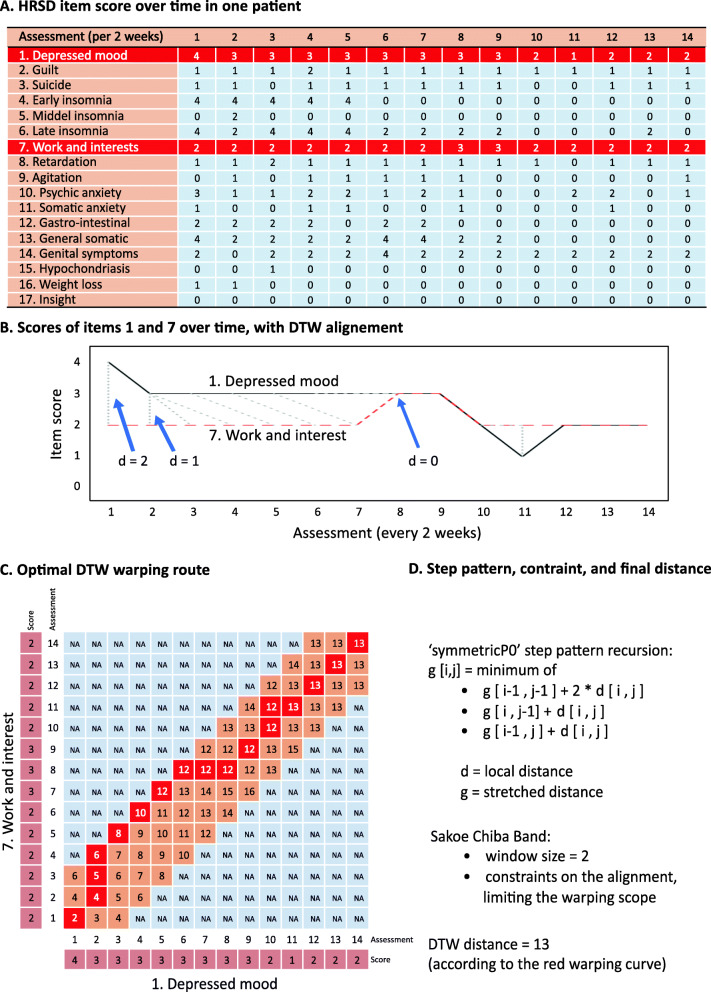
For a single patient, the individual HRSD-17 item scores over time are shown (**a**). The DTW method uses a dynamic (i.e., stretching and compressing) programming approach to minimize a predefined distance measure (e.g., Euclidean distance), in order for the two time-series to become optimally aligned through a warping path (**b**). The optimal warping route between items 1 and 7 is shown (**c**). Using the “symmetricP0” step pattern and a Sakoe-Chiba Band of 2, this yields a final DTW distance of 13 (**d**)

We first aimed to cluster individual symptoms based on the temporal features that they share *within each individual patient*. The clustering of symptom trajectories based on DTW consisted of two steps. First, the DTW distance between each pair of symptom trajectories was calculated. This is illustrated in Fig. 1 with the example of two HRSD-17 item time-series (item 1 “depressed mood” and item 7 “work and interest”) of a single patient. The temporal scoring (per 2 weeks) on the given items is seen in Fig. 1a, with the two items for which the DTW distance is calculated shown in red. This patient had 14 assessments over a period of 26 weeks. The trajectories of items 1 “depressed mood” and 7 “work and interest” over time are plotted in Fig. 1b. The deformations of the time axes between both items are added, which brings the two time-series as close as possible to each other, in which all elements must be matched. Next, the calculation of the shortest path between the two time-series is shown in Fig. 1c. The two time-series were aligned in time with compressions and expansions. The “symmetricP0” step pattern was used as the dynamic time warping algorithm to match the two sequences, resulting in the red “warping path.” A Sakoe-Chiba Band of 2 was used in order for the severity scores to be matched to a maximum of plus or minus two time points (plus or minus a maximum of 4 weeks). Resulting from the DTW method, a distance measure (*d*) is produced: items with the best alignment, having a more similar slope and other dynamics (i.e., changes that co-vary over time), resulted in the smallest distance. The distance measures of each of the 17 time-series of individual HRSD-17 items are grouped in a distance matrix, comprising (17^2^ − 17)/2 = 136 distances for each individual patient.

Second, this matrix of 136 distances was presented in a heatmap and used in a hierarchical cluster analysis and a symptom network per patient. For the hierarchical cluster analysis, each item is initially assigned to its own cluster, and then the algorithm proceeds iteratively, at each stage joining the two most similar clusters, continuing until there is just a single cluster. We assumed 3 clusters for each patient, for illustrative purposes only, to enable easier recognition of the symptom with the more similar trajectories. We excluded all symptoms with a score of 0 throughout follow-up, as these tended to cluster together most strongly as these symptom pairs will have a distance of 0. At each stage, distances between clusters are recomputed by the Lance-Williams dissimilarity update formula according to the “Ward.D2” clustering methods. With “Ward.D2,” the total within-cluster variance is minimized, and the dissimilarities are squared before cluster updating.

Using the “qgraph” package, the structure of the network based on the distance matrix was visualized per patient, providing another way of graphical presentation of the clusters. We followed the recommendations on network analysis written by the developers of the R package [39]. A network with up to 17 nodes (representing the individual HRSD-17 depression symptoms) is obtained and, connecting them, the edges representing the distances between symptom trajectories. The thickness of the edges indicates the strength of the longitudinal elastic covariation (thicker edges represent a shorter distance between the two symptom trajectories).

### Inter-individual approach

Next, we aimed to study the aggregated dynamics of individual symptoms to yield systematic patterns over time across patients. In this second part, we aimed to build a generalizable hierarchy of symptom clusters based on their shared temporal features. First, the 136 distances were averaged over the 255 patients, weighted for the number of assessments that were done for each of the patients (ranging from 2 through 17). Second, this matrix of 255 mean distances was used for the generalizable hierarchical cluster analysis. A scree plot was constructed displaying the heights in a downward curve and the elbow rule (i.e., the point where the graph leveled off) was used to determine the most appropriate number of clusters.

The “Distatis” algorithm from the “DistatisR” package was used to check whether using the actual 255 distance matrices instead of one mean distance matrix yielded similar clusters. Distatis is a generalization of classical multidimensional scaling (MDS), based on a three-way principal component analysis, to analyze a set of distance matrices. In order to compare these distance matrices, it combines them into a common structure called a compromise and then projects the original distance matrices onto this compromise. Compromise factors are calculated and plotted in the compromise space, with each component been given the length corresponding to its eigenvalues. We plotted each of the 17 HRSD symptoms on an X-Y plane according to their first and second compromise factor values.

In the following step, we investigated two centrality metrics, being closeness centrality and degree centrality [40] for the average distance matrix. Degree centrality is based on the number and strengths of connections each symptom has. Closeness centrality also takes the global network structure into account because it measures the average distance of a certain symptom to all other symptoms. Applied on the DTW data, closeness is inversely proportional to the mean DTW to all other symptoms and, in this way, indicates which symptom trajectory is the most similar to that of other symptoms.

Finally, we computed the average DTW distance among all symptom trajectories for each patient. Symptoms that scored consistently zero were deleted from these analyses for that particular patient, as all such symptoms would result in distances of zero. Shorter average DTW distances reflected denser interconnections between symptoms, and longer average DTW distances reflected looser longitudinal connectivity between symptoms. In order to investigate the relationship between network density and reaching response and remission, we calculated the residuals of the regression with the number of assessments and the HDRS sum score. These residuals were plotted using box plots according to whether response and remission were reached, and we performed Wilcoxon signed-rank tests to compare the two samples.

**Fig. 2:**
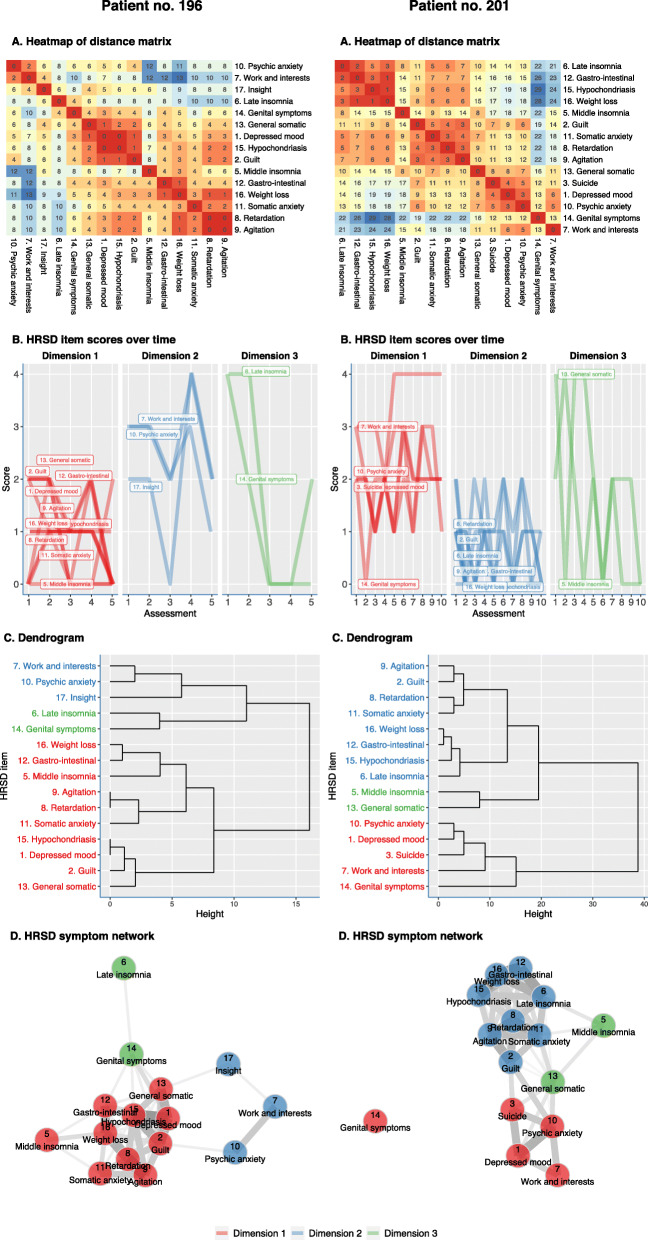
DTW analysis of HRSD-17 symptoms for patient no. 196 and patient no. 201 (**a**). Heatmap (symptoms that show high correlation are given a “hot” red color, and those that are not correlated are given a “cold” blue color) (**b**). Dendrogram based on the clustering of DTW distances of 15 of the non-zero HRSD-17 item scores over time (**c**). Network graph based on the distance matrix: connections between symptoms (edges) indicate distances between symptom trajectories (**d**). Centrality statistics of the network graph: centrality is based on the number and strengths of connections each symptom has. Closeness also takes the global network structure into account (**e**)

The analyses used the packages “dtw” (version 1.20.1), “pheatmap” (version 1.0.12), “parallelDist” (version 0.2.4), “qgraph” (version 1.6.2), and “DistatisR” (version) for the R statistical software (R version 3.6.0; R Foundation for Statistical Computing, Vienna, Austria, 2016). A sample code (with data from the of 2 exemplar patients of Fig. 2) can be found in Additional file 1.

## Results

### Patient characteristics

Table 1 shows the demographic and clinical characteristics and the use of psychotropic medication of the included patients. Patients had a mean age of 50.9 years (standard deviation [SD] = 15.4), and 165 were women (64.7%). A bipolar disorder was diagnosed in 48 patients (18.8%). The mean duration of illness was 11.2 ± 15 years. For 56 patients (22%), the current episode was the first depressive episode. The baseline HRSD-17 score was 20.7 (SD 4.6) on average, and 79.6% of the patients used antidepressants. Of the 255 patients, 169 showed treatment response and 128 remission at the end of admission. The median duration of hospitalization was 11 weeks, and the total number of assessments was 1480, with a mean of 5.8 and a median of 5 HRSD-17 assessments per patient.

**Table 1:** Characteristics and medication use of 255 consecutive depressed inpatients

Variable	Mean (SD)/no. (%)
**Demographic characteristics**	
Female (%)	165 (64.7)
Bipolar disorder	48 (18.8)
Age in years, mean (SD)	50.9 ± 15.4
Education^a,b^ (*n* (%))	
- Lower	41 (16.1)
- Intermediate	119 (46.7)
- Higher	93 (36.5)
Work status (*n* (%))	
- Unemployed	149 (58.4)
- Employed	93 (36.5)
- Others (student/voluntary service)	13 (5.1)
Marital status (*n* (%))	
- Married	109 (42.7)
- Divorced/widowed	65 (25.5)
- Never married	81 (31.8)
Living situation^c^ (*n* (%))	
- Living alone	76 (29.8)
- Living with partner	96 (37.6)
- Living with family	83 (32.5)
**Clinical characteristics**	
Index (first) depressive episode (*n* (%))	56 (22)
History of 4 or more depressive episodes (*n* (%))	76 (29.8)
Lifetime substance abuse/dependency^d^	
- Alcohol	27 (10.6)
- Drugs (THC, hard drugs, benzodiazepines)	5 (2.0)
Melancholic features	159 (62.4)
ROM baseline total scores	
- HRSD-17	20.7 ± 4.6
- BDI-II	33.7 ± 9.2
Baseline medication use	
- Antidepressants	203 (79.6)
- Antipsychotics	118 (46.3)
- Mood stabilizers	34 (13.3)
Responders (%)	169 (66.3)
Remitters (%)	128 (50.2)

Data are mean (SD) or no. (%), when appropriate

*ROM* routine outcome monitoring, *HRSD-17* 17-item Hamilton Rating Scale for Depression, *BDI-II* Beck Depression Inventory

^a^Lower education, general basic education only; intermediate education, middle vocational education; and higher education, higher vocational education or university

^b^Two missing values for education

^c^Living alone includes living in a home for the elderly and the convent

^d^Investigated using the MINI modules on substance abuse and suicidality

### Intra-individual approach

In Fig. 2, the DTW analyses of 2 exemplar patients are shown. We will discuss these two exemplar patients in order to demonstrate the opportunities of the DTW clustering method to inform clinical practice. By comparing the results from patients 196 and 201, we can already see a high degree of inter-individual variability in symptom trajectories.

Patient 196 was a 55-year-old female presenting with psychotic depression. At admission, anhedonia, insomnia, and psychic anxiety were overtly present. The anxious preoccupations disabled her in engaging any psychotherapy program at the start of hospitalization. A treatment with electroconvulsive therapy (ECT) led to a resolution of the most central symptoms (symptom with most dense connections with other symptoms, e.g., depressed mood, feelings of guilt, and somatic anxiety) during the hospitalization of 2 months. Although she remained to score relatively high on the HRSD-17 symptoms “work and interests,” “psychic anxiety,” and “insight,” she could be discharged after the resolution of the majority of her depressive symptoms. The central (red) symptoms tended to fluctuate most strongly together over time. Furthermore, due to the presence of residual symptoms that were resistant to ECT treatment, we formulated an advice for ambulatory psychological therapy to focus on these persistent (blue) symptoms of insight, engagement in activities, and psychic anxiety as a cornerstone of further treatment.

Patient 201 was a 38-year-old female who presented with a severely depressed mood and suicidal thoughts. She described her depressive complaints as an overpowering sense of feeling down and agitated. There was no loss of appetite or weight loss. A treatment with nortriptyline and trazodone (for her sleeping problems, mainly middle insomnia) was started. The sleeping problems improved quickly. Her depressed mood and suicidal thoughts did not change at the beginning of treatment. Treatment with lithium, because of a suspicion of underlying bipolar disorder, led to a quick resolution of the mood and anxiety symptoms. Loss of sexual interest was initially not present but commenced during hospitalization, possibly as a side effect of treatment.

### Inter-individual approach

**Fig. 3:**
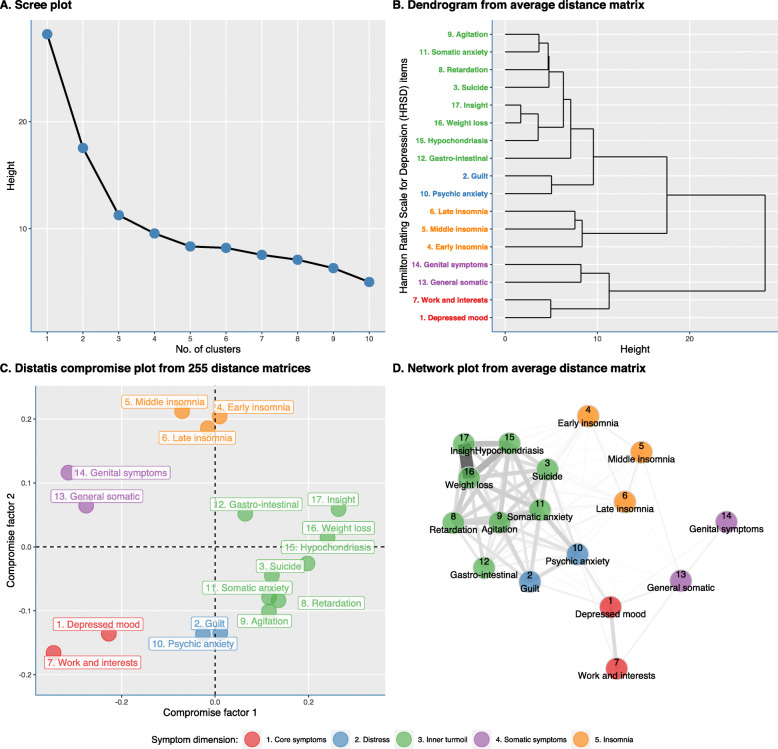
Nomothetic analyses based on all distance matrices from 255 depressed inpatients (see accompanying PDF). The scree plot displays the eigenvalues in a downward curve. The number of factors was determined using the elbow method (i.e., the point where the slope of the curve is leveling of; in our example, this is 5: after this point, the slope of the curve is nearly stable) (**a**). Ward’s (D2, i.e., general agglomerative hierarchical clustering procedure) clustering criterion on the weighted mean distance matrix from 255 patients (**b**). Distatis analysis: the PCA of the compromise matrix (i.e., weighted average of individual cross-product matrices) gives the position of the objects in the compromise space (**c**). Overview of the networks of HRSD-17 items for 255 patients (**d**)

Figure 3 shows the nomothetic analysis of the 255 patients. A total of five clusters emerged, based on the elbow method in the scree plot (see Fig. 3a). The hierarchical cluster analysis was estimated based on the average weighted distance matrix (Fig. 3b). These clusters consisted of symptoms with a similar course trajectory: (1) core symptoms (2 items: “depressed mood,” “work and interests”), (2) sleep symptoms (3 items: late, middle, and early insomnia), (3) distress (2 items: “guilt,” “psychic anxiety”), (4) somatic symptoms (2 items: “genital symptoms,” “general somatic symptoms”), and (5) inner turmoil (8 items: insight, weight loss, hypochondriasis, gastro-intestinal symptoms, somatic anxiety, agitation, retardation, and suicide). As is shown in Additional file 2: Fig. S1, the network plots did not change significantly when excluding all symptoms with a score of 0 throughout follow-up.

In the following step, we analyzed the actual 255 distance matrices, instead of one mean distance matrix, using Distatis. Figure 3c shows the Distatis compromise plot in which each HRSD-17 item is plotted according to their first and second compromise factor values. The distribution pattern of the HRSD-17 items in the compromise plot shows a comparable pattern to the obtained hierarchical clusters, corroborating the obtained five clusters. The clusters corroborated those found with the hierarchical cluster analysis on the average distance matrix. Next, the average distance matrix was visually presented in a network graph in Fig. 3d.

**Fig. 4:**
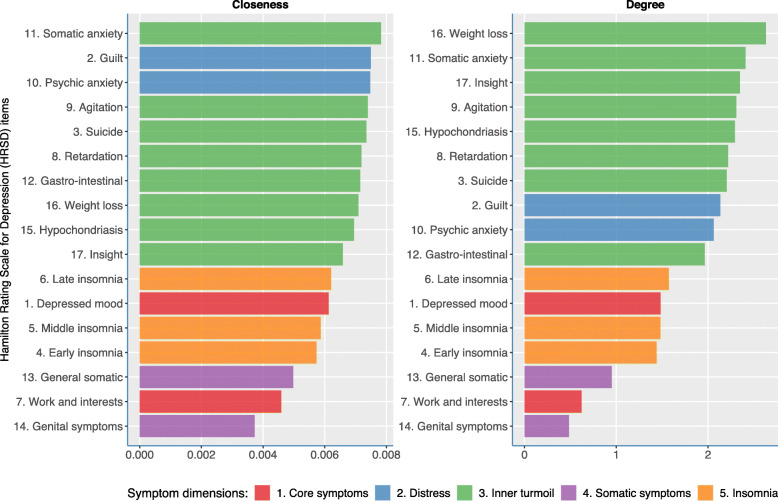
Centrality measures. The closeness centrality is the inverse of the average length of the shortest path between the focal node and every other node in the network (i.e., the more central a node is, the closer it is to all other nodes). Degree centrality represents the connectivity, based on the number and strengths of edges connected to it

Figure 4 shows the two centrality measures based on the network from the average distance matrix. These can inform us on which symptoms globally tend to covary together over time. The items from the “inner turmoil” show the highest degree centrality and closeness centrality scores, indicating that they covaried most strongly with other HRSD-17 items. Items constituting the “insomnia” or “somatic symptom” cluster showed lower centrality which suggests that these symptoms behaved in a more independent manner over time compared to the other HRSD-17 items.

**Fig. 5:**
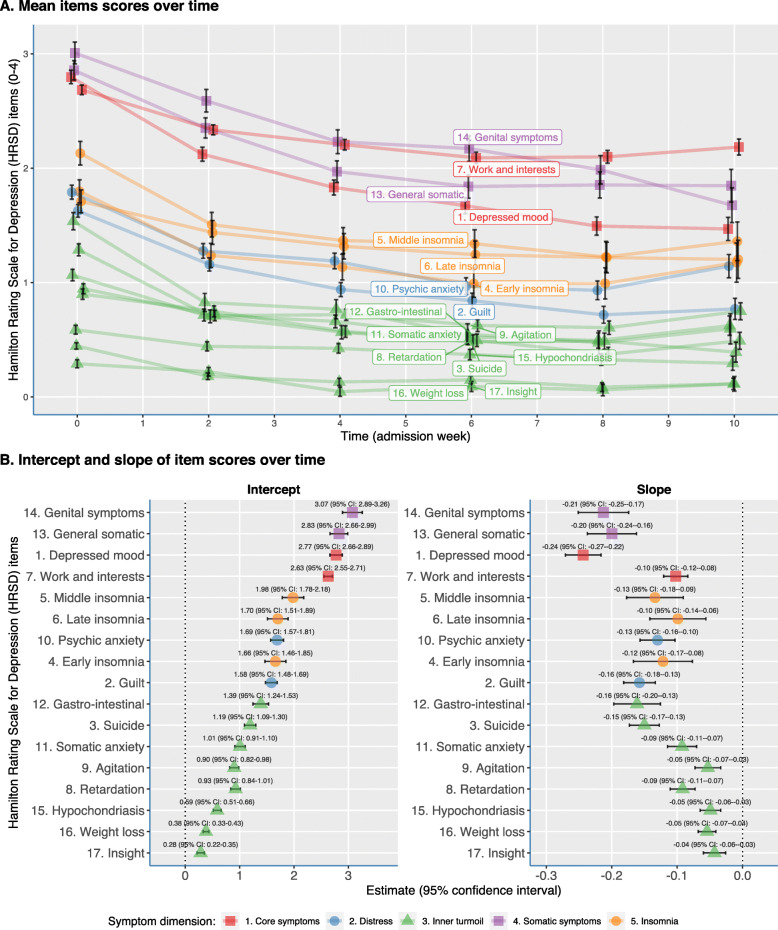
Forest plot of the 17 HRSD items of two mean levels of indicators of individual longitudinal trajectories. The mixed model intercept (**a**) indicates which symptoms scored the highest at baseline (i.e., genital, general somatic symptoms, and depressed mood scored the highest at baseline). The mixed model slope (**b**) of the linear model showed the average decline per 2-week time interval (i.e., depressed mood showed the steepest decline over time)

The evolution of the mean HRSD-17 item levels over time are visualized in Fig. 5a using mixed models per item. The eight items with a range from 0 to 2 (three insomnia items: gastro-intestinal complaints, general somatic and genital symptoms, insight and weight loss) were scaled to a range from 0 to 4 in order to make a comparability between all items possible. The HRSD-17 items “depressed mood,” “work and interests,” “general somatic,” and “genital symptoms” had the highest baseline severity. The items “insight” and “weight loss” had the lowest mean scores and stayed relatively low during hospitalization. Figure 5b shows the intercepts and slopes of the 17 mixed models for the individual longitudinal trajectories. The intercepts indicated that genital, general somatic, and depressed mood symptoms generally scored the highest at baseline. The slopes of the linear model revealed that depressed mood showed the steepest decline over time.

**Fig. 6:**
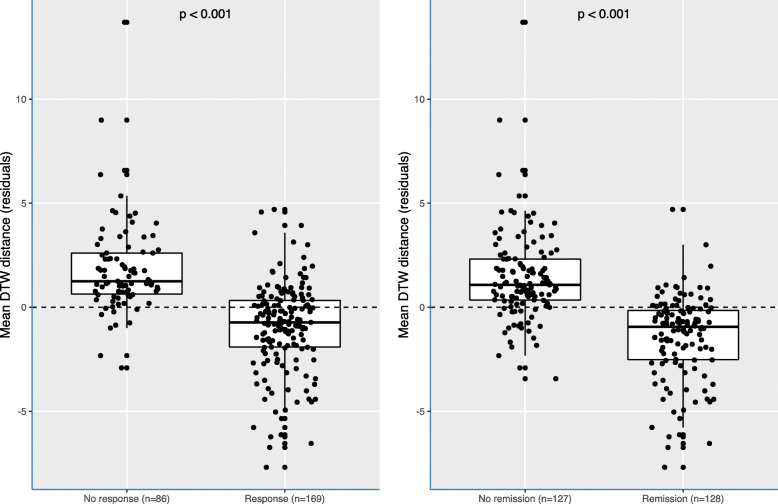
Average DTW distance according to response and remission. Those patients with response or remission had the shortest average distance among symptom trajectories, indicating denser interconnections (*p* by Wilcoxon signed-rank test to compare the two samples). Distances were adjusted for the number of assessments and the baseline total HRSD-17 score

As shown in Fig. 6, patients that reached response or remission during hospitalization had significantly shorter average distances among symptoms than patients who failed to reach response or remission. That is, patients reaching response or remission mostly had on average a more densely connected symptom network (based on the mean DTW analysis). We excluded symptoms that scored zero throughout the admission, yet when these symptoms were included, this resulted in similar findings (see Additional file 2: Fig. S2A). In addition, not adjusting for HRSD-17 total scores at baseline did not alter the results (see Additional file 2: Fig. S2B). Exploring the difference in network connectivity between unipolar and bipolar depressed patients revealed a denser symptom network in bipolar than in unipolar patients (see Additional file 2: Fig. S3).

## Discussion

The present study is the first to analyze the time-series of depression symptoms using DTW analyses in psychiatric inpatients. We applied the DTW computational method to estimate and visualize similarities in symptom trajectories and to yield clusters of symptoms with similar course trajectories both at the patient level and at the group level. Both the intra- and inter-individual analyses may help to increase our insight into the dynamical complexity of symptom trajectories in severely depressed inpatients. Furthermore, combining ROM techniques with automated feedback for the clinician based on the methods—as introduced here—proved useful to inform and facilitate clinical decision making. Overall, three major findings are worth discussing in more detail.

We first focused on the individual symptom dynamics that proved to be highly variable across individuals and thus idiosyncratic [41]. This finding supports previous concerns on the use of sum scores for assessing treatment outcome, since sum scores do not represent this dynamical symptom complexity well [4, 16], with a loss of substantial information that may be of clinical relevance. The intra-individual *dynamic* symptom clusters and symptom networks, in which the edges between symptoms represented the dynamical relation between them, allowed us to gain insight into the relative importance of certain symptoms for individual patients, but also at the group level [40]. Such symptoms may cause other symptoms, which may be different for other patients [9]. It could be hypothesized that targeting treatment on such central symptoms early in therapy may lead to a more rapid resolution of closely connected depressive symptoms [42, 43].

Overall, the study of intra-individual temporal dynamics of depression symptoms is rare in the literature. A growing field of research has focused on the development of individual dynamic networks of symptoms in which time-series or experience sampling methods (ESM) data are used to study the within-patient dynamical structure of symptoms [43–47]. These networks are mostly estimated using vector autoregression (VAR) which estimates both lagged (i.e., time minus one temporal) and contemporaneous (i.e., simultaneous) relationships among multiple symptoms [45]. The DTW approach represents a less constraint analysis of individual symptom dynamics since the DTW distance measure accounts for a longer time period when measuring the similarity between each pair of depressive symptoms (2 time points). Furthermore, it provides an accessible and easily interpretable method that can be a useful tool for clinicians and researchers for the early detection of central symptoms and the directed tailoring of treatment towards these symptoms. Moreover, it does not necessitate the time-consuming ESM data collection, which can be challenging in daily clinical practice due to the considerable burden it puts on participants.

Secondly, we focused on the group-level analyses, which yielded five symptom clusters with more similar dynamics over time across the total sample. Compared to cross-sectional factor analytic studies, which investigate the co-occurrence of depressive symptoms at a certain time point, we similarly found that the three sleep items appeared consistently in one factor [20, 21]. Previously, there was some support for the presence of a “general depression,” with depressed mood, guilt, suicide, work and interests, and psychic anxiety appearing on one factor [20–22]. We, however, found that only “depressed mood” and “work and interest” showed the most consistent trajectories over time, which represented the core symptoms of depression. Somatic symptoms did not appear on the same factor as described for cross-sectional factor analyses (“somatic symptom” or “somatized depression” consisting of somatic symptoms, weight loss/gastro-intestinal symptoms, loss of libido/genital symptoms) [20, 22]. Moreover, previous studies found evidence for limited longitudinal invariance, where the number of factors did not hold across time [24, 48] which is supported by our and previous data that the Omegas and Cronbach’s alphas were not stable over time, but improved during hospital admission [49]. An internal validation of our findings using a random sample of 128 and 127 patients of our sample revealed the same dynamical clusters (see Additional file 2: Fig. S4). Further validation of these findings in an independent sample is necessary.

Third, we found that patients who reached response or remission during hospitalization had a more densely connected symptom network compared to patients that failed to reach response or remission. This contrasts with the findings of Van Borkulo et al. [50], who identified a more densely connected cross-sectional depression symptom network in not remitters compared to patients reaching remission. Although the method of quantifying network connectivity was not the same (average DTW distance versus network comparison test), this shows, once again, the importance of studying longitudinal networks besides cross-sectional symptom networks. Our findings could be related to the literature reviewed by Scheffer [51], showing that networks with high connectivity can change more abruptly (for better and worse) in response to external events (so-called critical transitions). Applied on the DTW network analysis, when symptoms have a low level of connectivity, they seem to behave more independently from each other and in response to an external factor such as admission and treatment, which may have lowered the probability of an acute response or remission to treatment. These findings need to be confirmed in further studies, as the definition of response and remission was also based on the HRSD sum scores, which is not independent of the DTW assessments from HRSD time-series data.

The DTW method has a promising potential for clinical practice, and it builds further upon the already available evidence of the value of measurement-based care in psychiatry [52]. First, the DTW symptom clusters allow the clinician to gain insight into the dynamics of individual depression symptoms and longitudinal symptom clusters. Second, as illustrated in the two idiographic analyses (Fig. 2), the DTW method has the potential to facilitate clinical decision making. More specifically, treatment interventions targeted at the most central symptom (i.e., symptoms with the most dense connections with other symptoms) could lead to a rapid resolution of the depressive syndrome. Third, the graphical representation of the DTW clusters is easily amenable as a feedback tool for patients to gain more insight into the central symptoms that tend to covariate with a variety of other symptoms or in symptom clusters that tend to move in a more independent matter. This could lead to a more nuanced insight in reaching response or remission or lack thereof.

An important strength of our study is the use of the innovative DTW clustering method to study the time-series of individual symptom severity scores. The DTW method is able to process the highly dimensional ROM time-series data in order to reduce the complexity of the data while still maintaining the essential characteristics of the dataset. By using an elastic measurement, DTW provides an optimal time alignment between two time-series. Furthermore, DTW can be accurately used in smaller datasets and individual patients [31]. Another strength of our study is the relatively complete dataset of ROM data from real-world consecutive inpatients. Nonetheless, our results must be considered in light of some limitations. First, exclusively inpatients were recruited from one center which may limit the generalizability of our results to outpatients and other patient groups. Second, patients were treated with a variety of different combinations of psychotropic drugs which likely affected the course and dynamical characteristics of individual symptoms (such as concentration difficulties in those receiving ECT). Future studies using data from randomized trials may help to unravel the influence of different treatment strategies on the dynamic symptom dimensions. Third, the HRSD-17 is not designed to investigate individual symptoms, and its items are scored on a crude scale with only three or five answer categories resulting in low variability and precision. Fourth, assessments were done with 2-week intervals, and DTW analyses may be more useful in more frequent time-series like those collected with ESM. Fifth, the DTW method allows some flexibility in how it is applied to study MDD symptom trajectories, e.g., in terms of the global constraint (Sakoe-Chiba Band). We adopted default settings based on simulation studies in the prior literature and hope that future methodological studies working with psychiatric data specifically will investigate how robust empirical results are to changes in default settings of the DTW method, e.g., using multiverse analyses [53].

## Conclusion

MDD is a heterogeneous disorder consisting of dynamic symptom clusters that varied between patients. The use of repeated, standardized clinical rating scales yields extensive information on patient-specific symptoms dynamics. DTW may be a promising new methodology for the study of the complex dynamic system of interacting psychiatric symptoms [9, 15, 54] with the potential to facilitate personalized psychiatry care.

## Supplementary Information

**Additional file 1.** Sample R script for paper.

**Additional file 2: Figure S1.** Network plots based on the DTW analyses in the 255 patients. We compared the network structure based on all data from all patients, and the analyses based only on symptoms that did not score as 0 throughout the follow-up. Such symptom pairs with scores of 0 will result in a distance of 0, solely because they were absent in some patients. **Figure S2.** Average DTW distance according to response and remission, including symptoms that scored zero throughout the hospitalization. **Figure S3.** Network plots based on the DTW analyses in 231 of the 255 patients, who had a clinical diagnosis of either MDD of BD. We compared the network structure [A and B] and calculated the mean distance only on symptoms that did not score as 0 throughout the follow-up. Although the network structure was largely similar, on average patients with BD had a denser distance matric than patients with MDD (*P*=0.0028). **Figure S4.** Network plots of two subsamples [**A** and **B**] of the 255 patients. We used an automated split with a subset of 128 and 127 patients, in which we conducted separate DTW analyses. Node placement was done by using the Procrustes algorithm (from the R Package ‘networktools’), to aid the visual comparison between the two networks. As a result, configurations were brought into a similar space in which statistically meaningless differences were removed without changing the fit. This analysis showed that the network (based on each of the average distance matrixes were stable. The congruence coefficient was high at 0.994, when we compared both sets of compromise factors derived from each Distatis analysis.

## Data Availability

The datasets used analyzed during the current study are available from the corresponding author on reasonable request.

## Abbreviations

*d*: Distance measure
DTW: Dynamic time warping
ECT: Electroconvulsive treatment
HRSD-17: 17-Item Hamilton Rating Scale for Depression
MDD: Major depressive disorder
MDS: Multidimensional scaling
SD: Standard deviation
VAR: Vector autoregression
