# Supplementary material for: Understanding personalized dynamics to inform precision medicine: a dynamic time warp analysis of 255 depressed inpatients
Source: BMC Med. 2020 Dec 23;18:400. doi: 10.1186/s12916-020-01867-5 (PMC7756914; doi:10.1186/s12916-020-01867-5)
Supplement: Supplementary file 1 — Additional file 1. Sample R script for paper. [file 12916_2020_1867_MOESM1_ESM.docx]

# Sample R script for paper, entitled: "Understanding personalized dynamics to inform precision medicine:

# a dynamic time warp analysis of 255 depressed inpatients"

# Script by: Erik J. Giltay and Kaat Hebbrecht

# email: e.j.giltay@lumc.nl and Kaat.Hebbrecht@emmaus.be

# Date: July 2020

# Load packages into memory

library(dtw)

library(parallelDist)

library(pheatmap)

library(tidyverse)

library(ggrepel)

library(DistatisR)

# The 17 HRSD items

items <- c("1. Depressed mood", "2. Guilt", "3. Suicide", "4. Early insomnia",

"5. Middle insomnia", "6. Late insomnia", "7. Work and interests", "8. Retardation",

"9. Agitation", "10. Psychic anxiety", "11. Somatic anxiety", "12. Gastro-intestinal",

"13. General somatic", "14. Genital symptoms", "15. Hypochondriasis",

"16. Weight loss", "17. Insight")

# HRSD data for patient 196 (See figure 2)

dat196 <- data.frame(T0 = c(2, 2, 0, 0, 0, 4, 3, 1, 1, 3, 0, 0, 2, 4, 2, 0, 2),

T0 = c(2, 2, 0, 0, 0, 4, 3, 1, 1, 3, 1, 2, 2, 2, 1, 1, 2),

T0 = c(1, 1, 0, 0, 0, 0, 2, 1, 1, 2, 1, 0, 0, 0, 1, 0, 0),

T0 = c(2, 1, 0, 0, 0, 0, 4, 1, 1, 3, 1, 0, 2, 0, 2, 0, 4),

T0 = c(0, 0, 0, 0, 2, 2, 2, 0, 0, 1, 1, 0, 0, 0, 0, 0, 2))

row.names(dat196) <- items

# HRSD data for patient 201 (See figure 2)

dat201 <- data.frame(T0 = c(3, 1, 2, 0, 4, 2, 3, 1, 1, 2, 1, 2, 2, 2, 0, 1, 0),

T2 = c(2, 1, 2, 0, 0, 0, 3, 1, 0, 2, 0, 0, 4, 0, 0, 0, 0),

T4 = c(1, 0, 1, 0, 4, 0, 3, 0, 1, 1, 2, 0, 2, 2, 0, 0, 0),

T6 = c(2, 1, 2, 0, 4, 2, 2, 1, 1, 2, 1, 0, 0, 2, 0, 0, 0),

T8 = c(1, 1, 1, 0, 2, 0, 3, 0, 1, 1, 0, 0, 2, 4, 0, 0, 0),

T10 = c(3, 2, 2, 0, 0, 0, 3, 2, 1, 3, 1, 0, 0, 4, 0, 0, 0),

T12 = c(1, 1, 1, 0, 2, 0, 2, 0, 0, 2, 0, 0, 2, 4, 0, 0, 0),

T14 = c(3, 2, 3, 0, 0, 0, 2, 1, 1, 2, 2, 0, 2, 4, 0, 0, 0),

T16 = c(2, 1, 3, 0, 0, 0, 2, 1, 1, 2, 0, 0, 2, 4, 0, 0, 0),

T18 = c(2, 1, 1, 0, 0, 0, 2, 0, 0, 2, 0, 0, 0, 4, 1, 0, 0))

row.names(dat201) <- items

# Plot the HRDS items scores over time

plot_hrsd <- function (dat){

dat %>%

t() %>% # transpose the data

as.data.frame() %>% # transform it back from matrix into a dataframe

rowid_to_column() %>% # create a unique variable to indicate the time

gather (item, score, "1. Depressed mood":"17. Insight") %>%

ggplot(aes(rowid, score, color = factor(item, levels = items))) +

geom_line() + labs(x = "Time", color = "HRSD items:")

}

plot_hrsd(dat196)

plot_hrsd(dat201)

# If you aim to remove all items that only scored 0 throughout follow-up

dat_no_zero196 <- dat196[reduce(dat196, `+`) != 0,]

dat_no_zero201 <- dat201[reduce(dat201, `+`) != 0,]

# Calculate the distances between all pairs of items

# (in parallel using multiple threads to increase speed)

distance196 <- parDist(dat196 %>% as.matrix(), method = "dtw",

window.type = "sakoechiba", window.size = 2, step.pattern = "symmetricP0")

distance201 <- parDist(dat201 %>% as.matrix(), method = "dtw",

window.type = "sakoechiba", window.size = 2, step.pattern = "symmetricP0")

# Create the clustered heatmap

heatmap_hrsd <- function(dist){

pheatmap(dist %>% as.matrix(),

display_numbers = TRUE, border_color = FALSE,

clustering_method = "ward.D2", treeheight_row = 0,

treeheight_col = 0, color = colorRampPalette(brewer.pal(n = 7, name = "RdYlBu"))(100),

number_format = "%.0f", legend = FALSE)

}

heatmap_hrsd (distance196)

heatmap_hrsd (distance201)

# Hierarchical cluster analysis on the disctance matrix (of dissimilarities)

distance196 %>% hclust(method = "ward.D2") %>% as.dendrogram() %>% plot()

distance201 %>% hclust(method = "ward.D2") %>% as.dendrogram() %>% plot()

# Distatis analyses on data from both patients

distatis_data <- list(distance196 %>% as.matrix(), distance201 %>% as.matrix()) %>%

simplify2array()

testDistatis <- distatis(distatis_data)

testDistatis$res4Splus$F %>%

as.data.frame() %>%

rownames_to_column("item") %>%

dplyr::rename (f1 = "Factor 1", f2 = "Factor 2") %>%

ggplot(aes(f1, f2)) +

geom_point(size = 8, alpha = 0.6) +

geom_vline (xintercept = 0, linetype = "dashed") +

geom_hline (yintercept = 0, linetype = "dashed") +

labs(x = "Compromise factor 1", y = "Compromise factor 2") +

geom_label_repel(size = 4, aes(label = item), alpha = 0.78,

show.legend = F, segment.size = 0) +

scale_colour_brewer(palette = "Set1")
