# Supplementary material for: Understanding personalized dynamics to inform precision medicine: a dynamic time warp analysis of 255 depressed inpatients
Source: BMC Med. 2020 Dec 23;18:400. doi: 10.1186/s12916-020-01867-5 (PMC7756914; doi:10.1186/s12916-020-01867-5)
Supplement: Supplementary file 2 — Additional file 2: Figure S1. Network plots based on the DTW analyses in the 255 patients. We compared the network structure based on all data from all patients, and the analyses based only on symptoms that did not score as 0 throughout the follow-up. Such symptom pairs with scores of 0 will result in a distance of 0, solely because they were absent in some patients. Figure S2. Average DTW distance according to response and remission, including symptoms that scored zero throughout the hospitalization. Figure S3. Network plots based on the DTW analyses in 231 of the 255 patients, who had a clinical diagnosis of either MDD of BD. We compared the network structure [A and B] and calculated the mean distance only on symptoms that did not score as 0 throughout the follow-up. Although the network structure was largely similar, on average patients with BD had a denser distance matric than patients with MDD (P=0.0028). Figure S4. Network plots of two subsamples [A and B] of the 255 patients. We used an automated split with a subset of 128 and 127 patients, in which we conducted separate DTW analyses. Node placement was done by using the Procrustes algorithm (from the R Package ‘networktools’), to aid the visual comparison between the two networks. As a result, configurations were brought into a similar space in which statistically meaningless differences were removed without changing the fit. This analysis showed that the network (based on each of the average distance matrixes were stable. The congruence coefficient was high at 0.994, when we compared both sets of compromise factors derived from each Distatis analysis. [file 12916_2020_1867_MOESM2_ESM.docx]

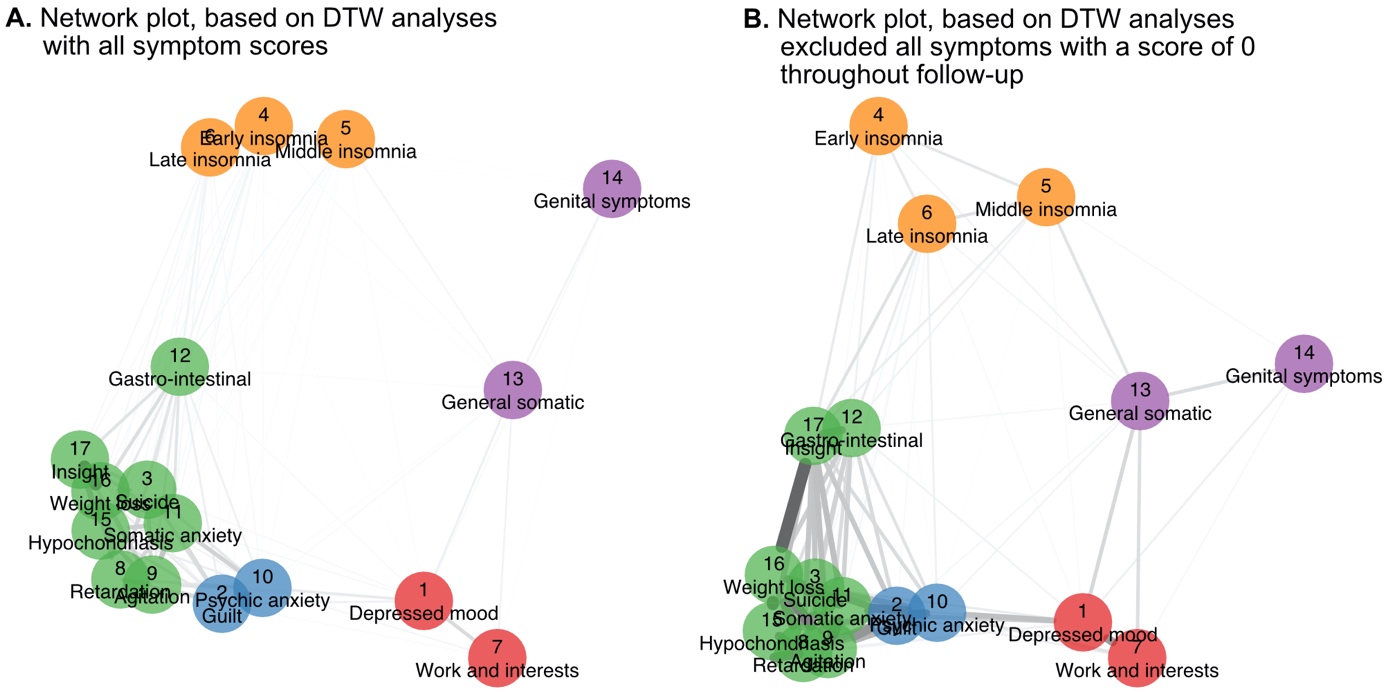


**Figure S1.**

Network plots based on the DTW analyses in the 255 patients. We compared the network structure based on all data from all patients, and the analyses based only on symptoms that did not score as 0 throughout the follow-up. Such symptom pairs with scores of 0 will result in a distance of 0, solely because they were absent in some patients.

**Figure S2.**

1. **Including all items (not excluded symptoms that scored zero)**


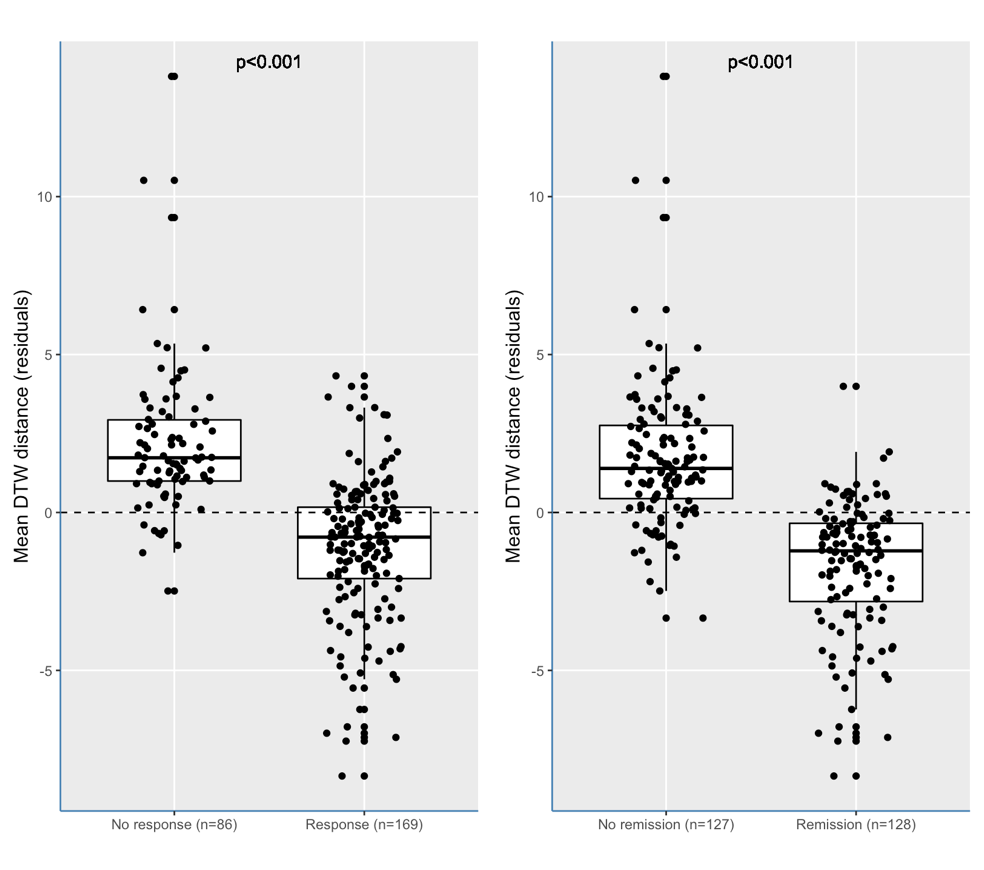


1. **Not adjusting for HRSD-17 total scores at baseline**


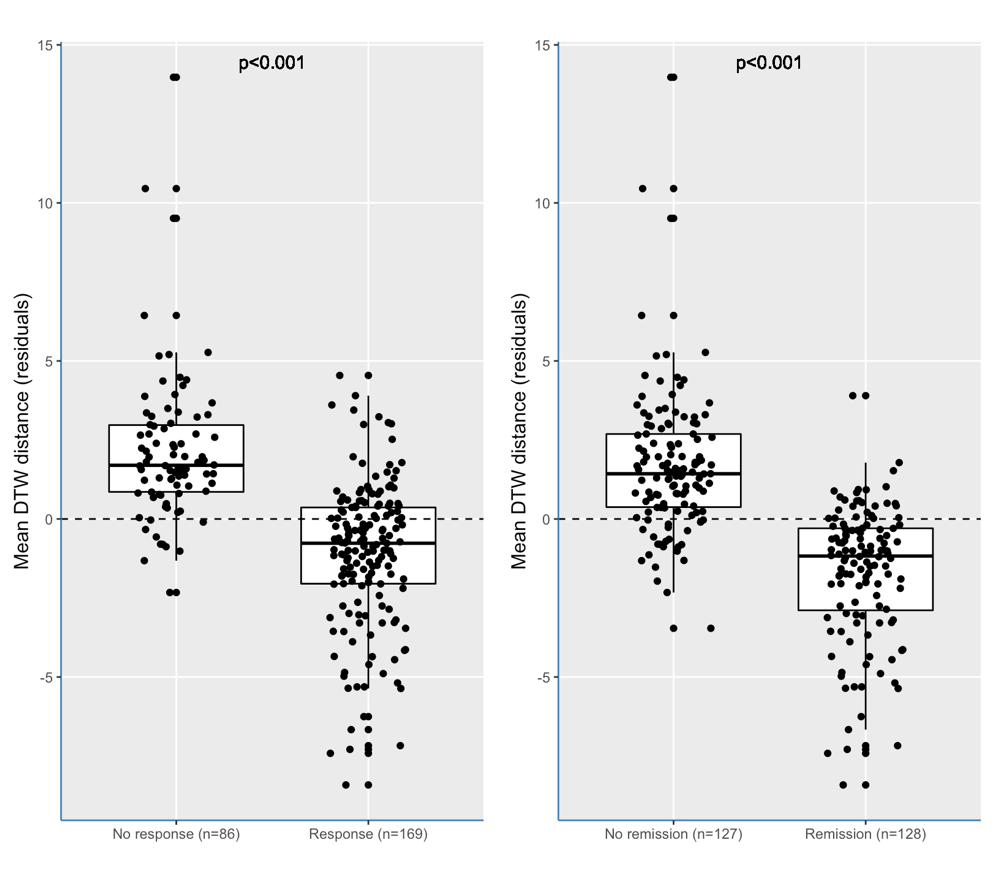


**
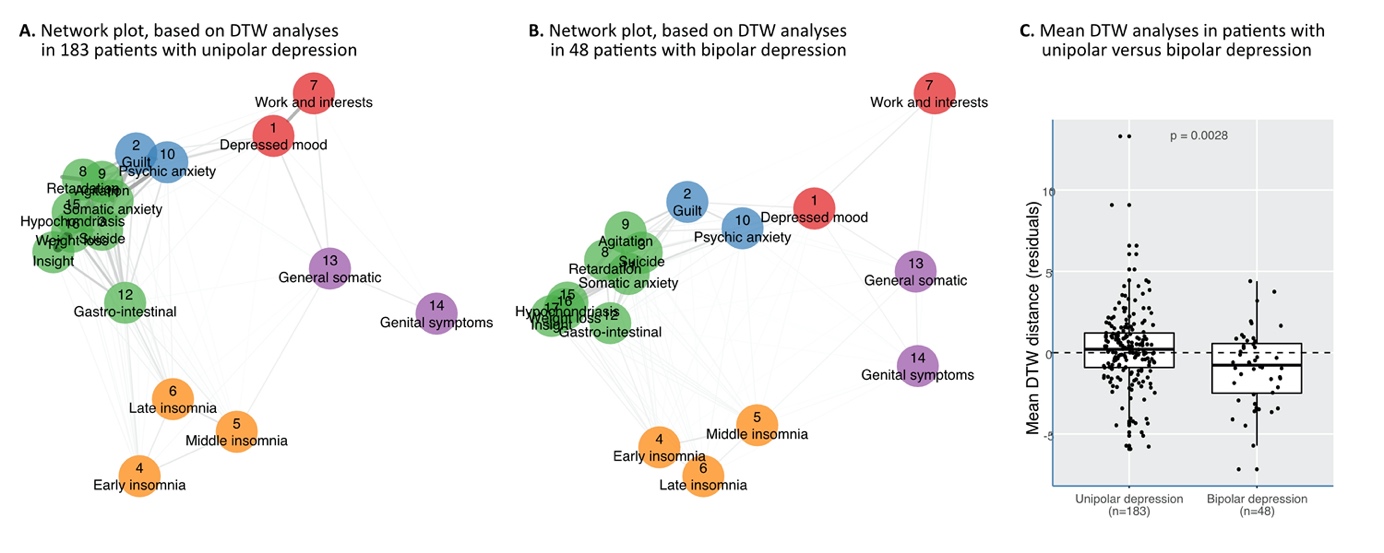
**

**Figure S3.**

Network plots based on the DTW analyses in 231 of the 255 patients, who had a clinical diagnosis of either MDD of BD. We compared the network structure [A and B] and calculated the mean distance only on symptoms that did not score as 0 throughout the follow-up. Although the network structure was largely similar, on average patients with BD had a denser distance matric than patients with MDD (P=0.0028).


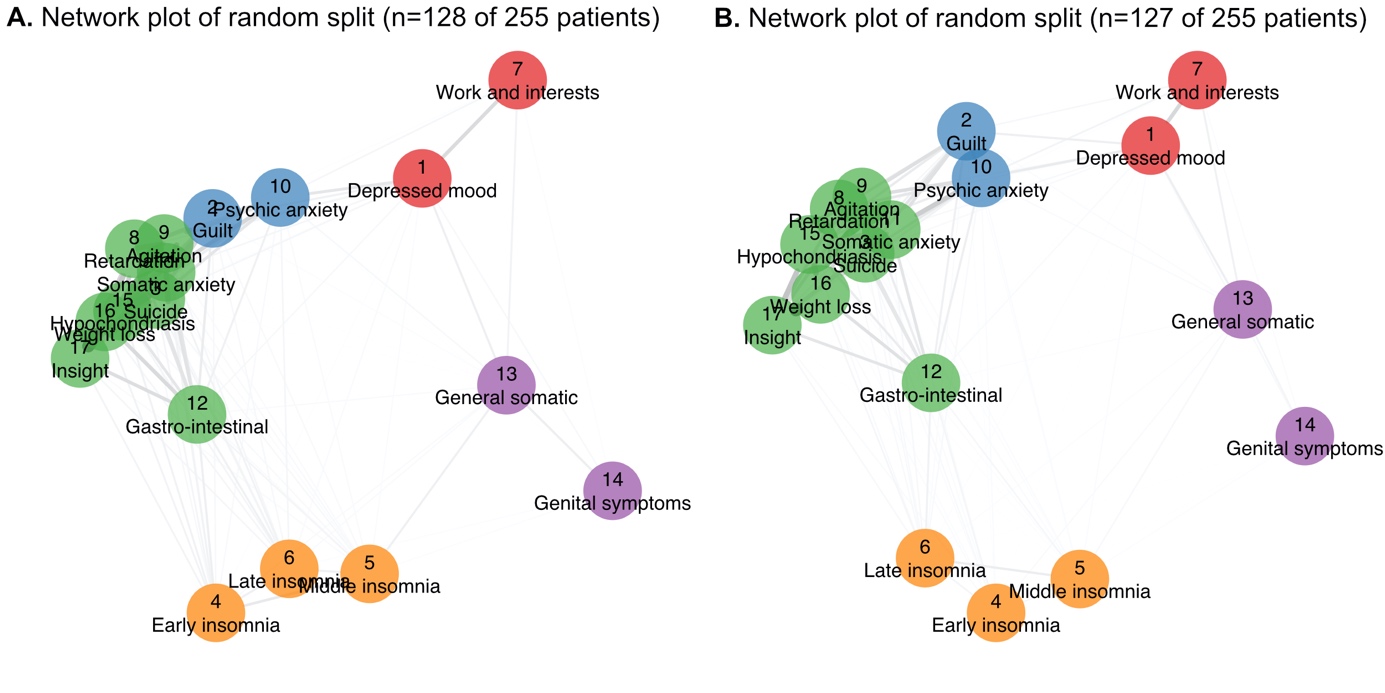


**Figure S4.**

Network plots of two subsamples [**A** and **B**] of the 255 patients. We used an automated split with a subset of 128 and 127 patients, in which we conducted separate DTW analyses. Node placement was done by using the Procrustes algorithm (from the R Package ‘networktools’), to aid the visual comparison between the two networks. As a result, configurations were brought into a similar space in which statistically meaningless differences were removed without changing the fit. This analysis showed that the network (based on each of the average distance matrixes were stable. The congruence coefficient was high at 0.994, when we compared both sets of compromise factors derived from each Distatis analysis.
